# 

**Published:** 1909-10-30

**Authors:** 


### The Question Box.

**SPECIAL NOTICE.**—Questions of interest affecting the honorary medical staff and hospital administration in all departments are invited. When any point arises we hope our readers will formulate it in a question and so co-operate to make this column of real value and interest. In this way the experience of the best-managed hospitals should be made helpful to the whole hospital world. We shall welcome the contribution of both questions and answers.

**N.B.**—The publication of an answer in this column does not necessarily preclude the publication of subsequent answers to the same question, for a question may involve points which require to be threshed out and fully discussed. Answers, if published, will be paid for at scale rates.

### VOLUNTARY HOSPITALS AND THE TICKET SYSTEM.

Question. Has the time arrived when each voluntary hospital where the ticket system prevails should make such tickets represent a subscription at least equal to the cost to the institution of the treatment given to the in- and out-patient.

ANSWER: No.

A Provincial View.

By "Discens."

No; for the following reasons:—

1. The ticket system in most hospitals is, at any rate so far as in-patients are concerned, very largely in abeyance.

2. Urgent cases are invariably admitted without ticket and on the doctors' recommendations.

3. The average cost of an in-patient is £5, and an out-patient, say, 2s. 6d. Subscribers of £1 1s. and £2 2s. like to have tickets by them in case of a request being made, even though they know tickets are not necessary in urgent cases. To adopt a "representative cost" basis would place in-patient tickets in the hands of the larger subscribers only and would alienate the one, two, and three guinea subscribers.

4. Many subscribers return or destroy their tickets. Only about one in ten in-patients' tickets issued are used. The law of average thus asserts itself, and renders it possible to provide tickets for small annual subscriptions.

5. Ministers of religion would be deprived of a very helpful asset in their work if the "representative cost" basis were adopted. Tickets are allocated to Church collections about half the rate of annual subscribers.

6. A proportion, more or less large, of the income of hospitals does not carry tickets—*e.g.* income from investments, donations, probationers' and patients' payments, miscellaneous receipts, legacies.

7. The limitations of hospital tickets would have the effect of making them very difficult to obtain by the very people who most need them, and neither the number of applications for treatment nor the extent of the work of the hospitals would be reduced.

Positive reasons will furnish good ground for discussion.

### POOR LAW INFIRMARIES.

Question. Should Poor Law Infirmaries have a visiting medical staff? If so, on what terms and conditions of attendance?

ANSWER: Not under existing conditions.

A Provincial View.

By Mr. J. F. Woodyatt, M.D., M.R.C.S., L.R.C.P., Medical Officer St. Luke's Hospital, Salterhebble, Halifax.

I am sorry to have been so late in sending you my views on Union Hospital Staffing. However, you shall now have what I think about the matter.

For a large up-to-date union hospital, that is, one with 400 beds or over (that is hospital beds without workhouse), I think by far the most suitable staff is a resident medical superintendent with a staff of assistants according to size, say one for each 200 beds. Of course the chief point turns on the suitability of the medical superintendent. I consider he should be a man of considerable hospital experience, both general and union, and well paid—at least £500 a year including emoluments. He should be able to do major surgery. This, I think, is a great advantage, both for the training of the nurses and experience of the residents. If the medical superintendent is unable to do major surgery, I think the Guardians ought to pay a good visiting surgeon to come in when called upon to operate in the hospital at a fixed salary, say £80 per annum.

If we are to have a visiting staff for large union hospitals, the conditions of appointment and service should be altered.

Large union hospitals should attract the best class of men, and then we are faced with the difficulty that the best men are busy men and not able to give the time to a union hospital that is absolutely necessary. Again, as union medical officers in hospitals remain in harness as a rule until sixty-five years of age, the hospital is liable to suffer from the medical officer, with advancing years, not being able either to give the time or being too old to do the work, or not keeping themselves up to date. These difficulties might be overcome by appointing the younger men, that is, those on the staff of the general hospital, for a period of years, say twelve, as in the Army and Navy, and granting them an honorarium at the end of their service in lieu of pension at sixty-five.

The visiting staff to any union should be a medical officer and a surgical officer, as at Crumpsall, Manchester, with two or more resident assistants as is thought necessary. In all cases the medical officers should attend in the mornings. In the smaller union hospitals the old-fashioned medical officer, I think, cannot be improved upon, except that he should retire before sixty-five and with a honorarium instead of pension. My experience teaches me that general practitioners over fifty have not the time or energy to look after union hospitals as they should be.

### GRADUATES' MEDICAL SCHOOLS.

DIARY FOR THE WEEK, NOVEMBER 1 to 6.

### THE POST-GRADUATE COLLEGE, West London Hospital, Hammersmith, W.

**At 10 a.m.—November 1** and **4**, Surgical Registrar, **Demonstration.**
**November 5**, Medical Registrar, **Demonstration.**
**At 12 noon.—November 4**, Dr. Bernstein, **Pathological Demonstration.**
**At 12.15 p.m.—November 2**, Dr. Pritchard, **Practical Medicine.**
**November 3** and **6**, Dr. Grainger Stewart, **Practical Medicine.**
**At 5 p.m.—November 1**, Mr. Rickard Lloyd, **Anæsthetics.**
**November 2**, Dr. Moullin, **Gynæcological Cases.**
**November 3**, Dr. Low, **Examination of the Blood in Tropical Diseases.**
**November 4**, Mr. Edwards, **Clinical Lecture.**
**November 5**, Dr. Abraham, **Cases of Skin Disease.**

### LONDON SCHOOL OF CLINICAL MEDICINE, Seamen's Hospital, Greenwich, S.E.

**At 4 p.m.—November 1**, Mr. Lake, **Post Nasal Catarrh.**
**At 2.15 p.m.—November 3**, Dr. Taylor.

### MEDICAL GRADUATES' COLLEGE AND POLYCLINIC, 22 Chenies Street, W.C.

**At 4 p.m.—November 1**, Dr. Graham Little, **Clinique (Skin).**
**November 2**, Dr. James Taylor, **Clinique (Medicine).**
**November 3**, Mr. A. Pearce Gould, **Clinique (Surgery).**
**November 4**, Sir Jonathan Hutchinson, **Clinique (Surgery).**
**November 5**, Dr. Jobson Horne, **Clinique (Ear, Nose, and Throat).**
**At 5.15 p.m.—November 1**, Dr. G. E. Herman, **Puerperal Eclampsia.**
**November 2**, Dr. Purves Stewart, **Facial Hemispasm.**
**November 3**, Dr. A. E. Giles, **The Menopause, Natural and Artificial.**
**November 4**, Dr. Leonard Guthrie, **Some Nervous Affections in Children.**

### NATIONAL HOSPITAL FOR THE PARALYSED AND EPILEPTIC, Queen Sq., Bloomsbury, W.C.

**At 3.30 p.m.—November 2**, Dr. Ormerod, **Tabes.**
**November 5**, Mr. Sargent, **Surgery of the Nervous System.**

### ST. JOHN'S HOSPITAL FOR DISEASES OF THE SKIN, Leicester Square, W.C.

Chesterfield Lectures.—At the Hospital on Thursday evenings at 6 p.m., each lecture followed by clinical demonstrations :—

**November 4, Syphilis :** History and Primary Invasion (Constitutional and Local). Eruptions, *Erythematous* : (I., Macular; and II., Maculo-Papular).

THE HOSPITAL October 30, 1909.

*Name* ..................................

*Address* ..................................

..................................

This Coupon must accompany manuscript or contributions intended for The Hospital.

